# Cognitive Vulnerability in Patients with Generalized Anxiety Disorder, Dysthymic Disorder and Normal Individuals

**DOI:** 10.5539/gjhs.v8n8p8

**Published:** 2015-12-17

**Authors:** Fateme Moin Al-Ghorabaie, Azam Noferesti, Mahdi Fadaee, Nima Ganji

**Affiliations:** 1 Faculty Member of Cognitive Science Research Group of Academic Center of Education, Culture and Research, Alborz Branch, Tehran, Iran; 2 Clinical Psychology, Tehran, Iran

**Keywords:** cognitive vulnerability, response styles, dysthymic disorder, generalized anxiety disorder

## Abstract

**Aim:**

The purpose of this study was to assess cognitive vulnerability and response style in clinical and normal individuals.

**Method:**

A sample of 90 individuals was selected for each of the 3 groups of Generalized Anxiety disorder, Dysthymic disorder and normal individuals. They completed MCQ and RSQ.

**Results:**

Results analyzed by MANOVA and post hoc showed significant differences among groups. Dysthymic group and GAD reported higher scores on cognitive confidence compared to the normal group. Individuals with GAD showed highly negative beliefs about need to control thought, compared to the other groups, but in cognitive self-consciousness they have no differences with the normal group. In regard to uncontrollability, danger and positive beliefs, GAD group had higher levels than the other groups. Although normal and GAD group didn’t show any significant differences in response style, there was a significant difference between Dysthymic group and other groups in all response styles.

**Discussion:**

Beliefs and meta-cognitive strategies can be distinguished between clinical and non clinical individuals. Also, findings support the Self-Regulatory Executive Function model.

## 1. Introduction

Cognitive disorders such as Dysthymic Disorder or GAD are among serious problems that could have consequences and implications in different aspects of person’s life. The concept of metacognition, defined as an individual’s experience and knowledge of his or her own cognitive processes, has been considered as an effective model in explaining cognitive disorders. According to Wells (2009), metacognition describes a range of related elements involved in the process of interpretation, verification or cognitive control. Wells (1995, 2002) believes that metacognition can play a key role in establishment continuation and treatment of anxiety disorders such as GAD. People suffering from GAD use worrying as a strategy to cope with their perceived threats. On the other hand, other researches of psychological disorders has attempted to conceptualize response styles; meaning that in dealing with negative mood, people could respond in two different styles, ruminative or distracting (Lyubomirsky & Nolen-Hoeksema, 1993). Ruminative response intervenes with instrumental behaviors and problem resolutions; moreover, ruminative responses, as a new factor in explanation of the effects of therapy and causes of anxiety disorders, have attracted the focus of experts in the field of cognitive behavior (Lyubomirsky & Nolen-Hoeksema, 1993). Furthermore, ruminative response and worrying are considered as predictors of negative mood, which has been confirmed by Segerstorm, Tsao, Alden and Craske (2000).

Considering the unpleasant outcomes of anxiety disorder (Bond & Dryden, 2002), before attempting to perform any model of psychotherapy, it is necessary to assess metacognitive mechanisms and processes involved in these disorders. Therefore, this study aims at identifying the differences in metacognitive beliefs and respons styles of people suffering from Dysthymic Disorder, GAD and normal individuals.

## 2. Method

### 2.1 Participants

The clinical samples of this study were selected through a targeted sampling from individuals referred to psychiatry and psychology clinics in 2012 (each group N=30). In order to control unwanted variables and to avoid difficulties in results interpretation, participants all had to fit the following criteria: No prior psychotherapy; No personality disorder; No usage of prescription drugs, and at least middle school education level. The non-clinical samples fit the same criteria and were randomly chosen from healthy people in Tehran.

## 3. Measures

### 3.1 Metacognitive Questionnaire (MCQ-30)

Wells and Cart Wright-Hatton (2004) introduced short MCQs containing 30 questions and 5 subscales in order to assess individual differences in positive and negative beliefs about worrisome and unwanted thoughts, metacognitive control and judgement about cognitive efficiency. According to Cronbach’s Alpha, stability of each subscale is as follows: Cognitive confidence (0.93), positive beliefs (0.92), cognitive self-consciousness (0.92), uncontrollability and danger (0.91), need to control thoughts (0.72) and overall score (0.93). This test also has a good convergence and validity.

### 3.2 Response Style Questionnaire (RSQ)

Nolen Hoeksema and Morrow designed this questionnaire to account for and assess reactions of people to negative mood and also to assess the extent of ruminativity of people with negative mood. This questionnaire includes 22 items for assessment of three different styles of responses (depressing, thinking and brooding) with a good internal consistency (alpha=0.89). It also has a good validity in predicting depression (Robinson & Alloy, 2003).

## 4. Findings

The results of variance analysis show a significant difference in all metacognitive beliefs between the three groups of participants.

As seen in Table 1, there is a significant difference between normal group and GAD, DD groups in cognitive confidence scale. It is apparent that the scores of GAD and DD groups are higher than those of the normal individuals.

**Table 1:** Bonferroni post hoc test results for comparing groups in subscales of MCQ and RSQ subscales

	Group 1	Group 2	MD	Sig.	result
Cognitive confidence	normal	GAD	00.4-	001.0	1<2
		DD	86.2-	005.0	1<3
Need to control	normal	GAD	60.2-	001.0	1<2
		DD	23.3	001.0	3<1
Self-consciousness	GAD	DD	83.5	001.0	3<2
	normal	DD	80.3	001.0	3<1
	GAD	DD	13.4	001.0	3<2
Depressing style	normal	DD	73.12-	001.0	1<3
	GAD	DD	66.10-	001.0	2<3
Thinking style	normal	DD	93.4-	001.0	1<3
	GAD	DD	43.5-	001.0	2<3
Brooding style	normal	DD	66.4-	001.0	1<3
	GAD	DD	26.4-	001.0	2<3

Group 1: normal; Group 2: GAD; Group 3: DD.

Considering the need to control scale, the differences of means between three groups are significant. The scores of GAD group are higher than normal individuals and this group scored higher than dysthymic group.

The significant differences between three groups in all response styles are as follows: the mean for dysthymic group is the highest and there are no significant differences between normal individuals and GAD group in all response styles.

According to Table 2, there is a significant difference between normal individuals and GAD group and also between GAD group and Dysthymic group. But no significant difference was observed between the scores of normal individuals and those of the Dysthymic group. Finally, based on the findings, we can confirm the hypothesis stating that there is a significant difference between groups under study about metacognitive beliefs and response styles.

**Table 2:** Duncan C test results for comparing groups in positive beliefs and uncontrollability subscales

variable	Group 1	Group 2	MD	Sig.	result
Positive beliefs	normal	GAD	30.3-	05.0	1<2
	GAD	DD	70.5	05.0	3<2
uncontrollability	normal	GAD	23.4-	05.0	1<2
	GAD	DD	86.5-	05.0	3<2

## 5. Discussion

Metacognitive theory suggests that dysfunctional beliefs about cognition, which forms metacognition, play a key role in establishment and continuation of clinical problems. In particular, negative ideas about uncontrollability and danger lead to a dominant pattern of thought, in which worrying and anxious thoughts are considered controlling elements and as a result, they lead to fixation of negative anxieties in an individual (Wells, 2000, 2009).

The findings of this study revealed significant differences between both clinical groups and normal individuals in cognitive confidence, meaning that higher scores in clinical groups lead demonstrates a reduction in their confidence in capabilities of their memory. Therefore, those who cannot trust their own minds to take preventive and subsequent actions have a feeling of uncertainty about themselves and environment and finally feel an increased necessity to control their mental processes. These finding are in agreement with other studies showing that depressed and anxious people who exhibit low cognitive confidence, express their anxious and depressed mood more excessively upon facing stressful situations. They elevate their own anxiety by undermining the ability to confront threats or daily challenges (Yilmaz, Gensoz, Davis, & Valentine, 2000). As a result, lack of cognitive confidence can be considered a metacognitive vulnerability factor in creating anxiety or depression (Yilmaz et al., 2011).

Other findings indicate significant differences between clinical and normal groups in self-consciousness and need to control subscales. Anxious and depressed individuals tend to more frequently self-validate their inner thoughts and also tend to control their thoughts more than normal individuals. Since negative thoughts or interpretations are products of negative beliefs stored in long term memory, it seems that people actively validate their beliefs through their own inner rules. These finding concur with metacognitive theory which believes metacognitive beliefs, through attempts to control worrying and negative mood and constant validation of them, lead to loss of resources for confrontation and harm the process of control in overall (Wells, 2000, 2009).

Significant differences between positive and negative beliefs (uncontrollability and danger) in clinical group, and especially higher scores in GAD compared to normal individuals, demonstrates causality role of metacognition in development of anxiety disorders, as earlier studies have shown that metacognitive ideas about uncontrollability and worrying about danger are good predictors of anxiety and depression (Yilmaz et al., 2011). According to Wells, Welford, King, Papageorgiou, Wisely & Mendle (2010) people suffering from anxiety disorders also suffer from their numerous worries. And their negative view about worries, also have positive and negative beliefs about those worries. But it seems that simultaneous activation of these beliefs put them into an impasse that they want to worry and at the same time stop worrying. This contradiction makes these patients strongly believe that worrying helps them to keep themselves and their relatives away from possible dangers; which in turn lead to a strong need to control, even more than seen in people suffering from obsessive disorders or depression. While a new study by Prados (2011) shows that, unlike earlier studies, positive beliefs about worrying does not play any role in establishing and continuing those worries, this study alone cannot revoke Wells’ theory. In people with symptoms of depression, negative beliefs about uncontrollability and danger can also be an indicator of depression in future stages of the illness (Williams et al., 1997).

On the other hand, the findings of this study show significant differences between dysthymic group and normal individuals in response styles. These findings confirm the Response Style theory of Nolen Hoeksema (1993), in which one method of identifying people prone to depression is by mental rumination in responding to daily sadness. Depressed people tend to ruminate their thoughts in order to increase their current state toward the more ideal state. This kind of response lingers and aggravates the low mood periods. An individual’s tendency to respond depressingly and his ruminant thoughts can be considered as fixed characteristic, since many studies have confirmed the effect of rumination in mood prediction. Therefore, this style of response results an elevated low mood even among the non-clinical subjects, and it can also predict the start and intensity of depression (Donaldson, Lam, & Mathews, 2004).

Nolen Hoeksema and Marrow (1993) also showed that depressed responses can predict the intensity of anxiety disorder after a strong stressful event. People suffering from intensive mood symptoms cannot deviate their focus from their depressed mood. This means that focus deviation activities are the results of inner elements such as deviant response style and therefore, are not derived from a motivational factor (Sakamoto, Kabbara, & Tanno, 2001). Most of the studies on Response Style theory have confirmed these response styles as inner tendencies in low mood (Nolen & Mooro, 1993; Lyubomirsky & Nolen, 1993; Watking, Teasdale, & Williams, 2000; Lam, Smith, Checkley, Rijsdijk, & Sham, 2003). Overall findings of this study are along with research literature on cognitive mechanisms in anxiety disorders and their shortcomings in clinical sample in comparison with other non-clinical sample; These cognitive mechanisms can help experts involved in therapy choose and use effective strategies in problematic areas.
